# Regulation of Mitochondrial Biogenesis as a Way for Active Longevity: Interaction Between the Nrf2 and PGC-1α Signaling Pathways

**DOI:** 10.3389/fgene.2019.00435

**Published:** 2019-05-14

**Authors:** Artem P. Gureev, Ekaterina A. Shaforostova, Vasily N. Popov

**Affiliations:** ^1^Department of Genetics, Cytology and Bioengineering, Voronezh State University, Voronezh, Russia; ^2^Voronezh State University of Engineering Technologies, Voronezh, Russia

**Keywords:** mitochondrial biogenesis, Nrf2, PGC-1α, aging, active longevity

## Abstract

Aging is a general degenerative process related to deterioration of cell functions in the entire organism. Mitochondria, which play a key role in energy homeostasis and metabolism of reactive oxygen species (ROS), require lifetime control and constant renewal. This explains recently peaked interest in the processes of mitochondrial biogenesis and mitophagy. The principal event of mitochondrial metabolism is regulation of mitochondrial DNA (mtDNA) transcription and translation, which is a complex coordinated process that involves at least two systems of transcription factors. It is commonly believed that its major regulatory proteins are PGC-1α and PGC-1β, which act as key factors connecting several regulator cascades involved in the control of mitochondrial metabolism. In recent years, the number of publications on the essential role of Nrf2/ARE signaling in the regulation of mitochondrial biogenesis has grown exponentially. Nrf2 is induced by various xenobiotics and oxidants that oxidize some Nrf2 negative regulators. Thus, ROS, in particular H_2_O_2_, were found to be strong Nrf2 activators. At present, there are two major concepts of mitochondrial biogenesis. Some authors suggest direct involvement of Nrf2 in the regulation of this process. Others believe that Nrf2 regulates expression of the antioxidant genes, while the major and only regulator of mitochondrial biogenesis is PGC-1α. Several studies have demonstrated the existence of the regulatory loop involving both PGC-1α and Nrf2. In this review, we summarized recent data on the Nrf2 role in mitochondrial biogenesis and its interaction with PGC-1α in the context of extending longevity.

## Introduction

Mitochondria play a key role in the pathogenesis of many diseases (Wallace, 2005; Lin and Beal, 2006; Lindblom et al., 2015; Ratliff et al., 2016; Boengler et al., 2017) and aging (Jang et al., 2018). The number of mitochondria and the amount of mtDNA rapidly increase in the fetus, starting from the blastocyst stage (Pikó and Taylor, 1987) and continuing to grow over the entire period of organism development and maturation (Heerdt and Augenlicht, 1990). However, there is still no consensus on what happens to the number of mitochondria with aging, since the data obtained in different tissues, at different stages, and with different methods are extremely controversial.

A number of authors report an age-related increase of the amount of mtDNA in skeletal muscle (Barrientos et al., 1997; Onyango et al., 2010). Studies based on electron microscopy have not shown any age-related changes in the number of mitochondria in skeletal muscle (Mathieu-Costello et al., 2005; Callahan et al., 2014). However, Corsetti et al. (2008) showed a decrease of the number of mitochondrial copies in skeletal muscles of aged mice. Studies by Kerner et al. (2001) and Chabi et al. (2008) showed a decrease of the marker mitochondrial enzymes activity in skeletal muscle, and the authors concluded that the number of mitochondria decreases in aging. Opposite conclusions made by various research groups may be associated not only with different methods for determining the number of mitochondria, but also with structural, biochemical and functional heterogeneity of skeletal muscles (Hepple, 2014). Studies using electron microscopy showed a decrease of the number of mitochondria in the heart (Tate and Herbener, 1976; Corsetti et al., 2008), whereas others did not reveal any changes (Schmucker and Sachs, 1985). However, there is no doubt that the area of the inner mitochondrial membrane decreases with age in heart (Sachs et al., 1977; Riva et al., 2006). In the brain, age-related decrease of the number of mitochondria was found using electron microscopy (Burns et al., 1979), enzymatic methods (Genova et al., 1997; Lenaz et al., 2000; Dencher et al., 2007) and estimation of expression of genes encoded by mtDNA (Ojaimi et al., 1999; Gureev et al., 2016).

We should emphasize that it is not only the number of mitochondria that is important, but also their functional state, which depends on mitochondrial biogenesis and dynamics, including fission/fusion and mitophagy. The dysregulation of these processes leads to age-related decrease in mitochondrial volume density and oxidative capacity per mitochondrial volume (Conley et al., 2000). The coordination between these processes is controlled by several mutually regulated signaling cascades, one of the most important being the Nrf2/ARE cascade (Holmström et al., 2016). Despite a growing interest in this signaling pathway, there are only a few reviews on the key role of Nrf2 in mitochondrial biogenesis and its interactions with the more studied “master regulator of mitochondrial biogenesis” PGC-1α (Ryoo and Kwak, 2018).

## Regulation of Mitochondrial Biogenesis by the PGC-1α-Signaling Cascade

Mammalian mtDNA is a circular DNA molecule of approximately 16.5 kb that possesses its own translational/transcriptional system, including 2 rRNA genes and 22 tRNA genes. It also has the non-coding region, the so-called D-loop, that contains the mtDNA replication origin and the transcription initiation site (Clayton, 2000).

Replication is performed by the mtDNA polymerase γ (POLG) consisting of the catalytic subunit encoded by the *POLG* gene and auxiliary dimeric subunit encoded by the *POLG2* gene (Graziewicz et al., 2006). mtDNA is transcribed by the mitochondrial RNA polymerase POLRMT (Tiranti et al., 1997). The key enhancer protein is TFAM (transcription factor A, mitochondrial), which ensures mtRNA unwinding and flexing required for the POLRMT binding to the mtDNA promoters. TFB2M (transcription factor B2, mitochondrial) acts as a specific dissociation factor that provides interaction between POLRMT and TFAM. Both TFB1M and TFB2M bind rRNA dimethyltransferases and, therefore, can function as rRNA modifiers (Rebelo et al., 2011). It was suggested that the major role of TFB1M is rRNA methylation and not its transcription factor function (Metodiev et al., 2009).

Nuclear respiratory factors NRF1 and NRF2 regulate expression of the electron transfer chain (ETC) subunits encoded by the nuclear genome (Evans and Scarpulla, 1990) and bind to the promoters of genes involved in mtDNA transcription. NRF1 binds to the specific promoter sites and regulates expression of TFAM (Virbasius and Scarpulla, 1994), TFB1M, and TFB2M (Gleyzer et al., 2005). Besides, nuclear respiratory factors, in particular NRF2, regulate expression of other mitochondrial enzymes, e.g., TOMM20 (translocase outer mitochondrial membrane), a key enzyme in the mitochondrial membrane transport (Blesa and Hernández-Yago, 2006). In turn, NRF1 and NRF2 are regulated by transcription coactivators, the most studied of which is PGC-1α (Scarpulla, 2008).

PGC-1α was discovered as a coregulator of PPARγ expressed in the brown fat at low temperatures that mediates adaptive thermogenesis (hence the name PGC-1α—**P**PAR-**G**amma-**C**oactivator-**1α)**. Later, it was found that PGC-1α acts as a coactivator for a much larger number of genes. It was demonstrated that PGC-1α interacts with both NRF1 and NRF2. Deletion of the *N*-terminal fragment in NRF1 abolishes the PGC-1α effect on mitochondrial biogenesis (Wu et al., 1999).

**FIGURE 1:**
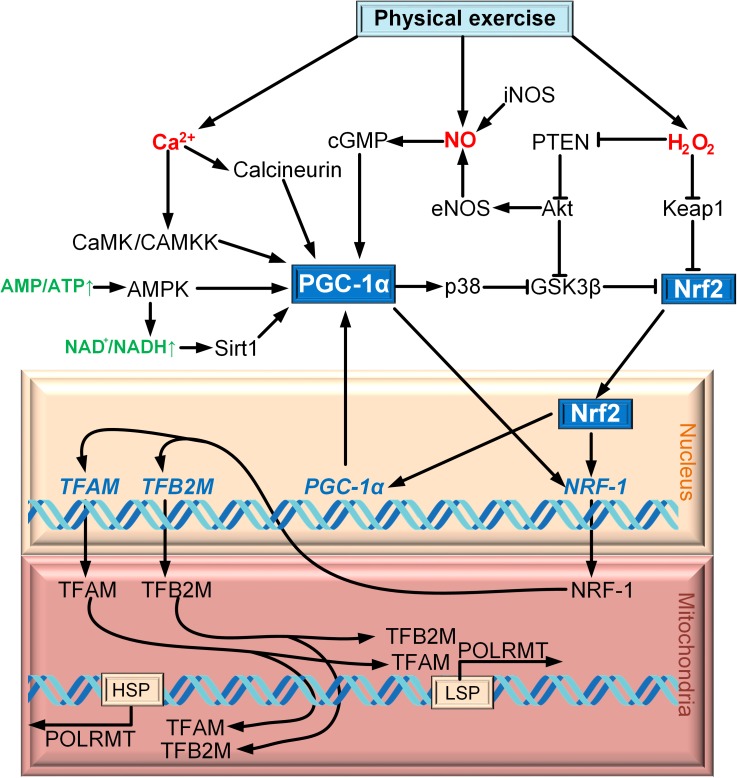
Scheme of interaction between Nrf2 and PGC-1α on the example of exercise.

PGC-1α is regulated on both the transcription and post-translation levels (Fernandez-Marcos and Auwerx, 2011). Cold exposure activates the sympathetic nervous system through β3-adrenergic receptor (β3-AR), which contributes the activation of protein kinase A (PKA). PKA activates CREB, which regulates the expression of PGC-1α directly (Puigserver et al., 1998; Boss et al., 1999; Herzig et al., 2001). p38 MAPK is another factor that regulates the expression of PGC-1α. It activates myocyte enhancer factor 2 (MEF2), which has the site of binding with the promoter of PGC-1α (Handschin et al., 2003). MEF2 can activate p38 mitogen-activated protein kinase (p38 MAPK) in skeletal muscle (Zhao et al., 1999). Additionally, p38 MAPK can regulate the expression of PGC-1α by activating transcription factor 2 (ATF2) (Akimoto et al., 2005). An increase in the intracellular Ñà^2+^ concentration in skeletal muscle activates Ñà^2+^/calmodulin-dependent protein kinase (CaMK), which activates the expression of PGC-1α via CREB (Ojuka, 2004) (Figure 1).

AMP-activated protein kinase (AMPK) is the major factor of PGC-1α post-translation modification for a wide range of tissue. PGC-1α phosphorylation is only one of the mechanisms of mitochondrial biogenesis activation via AMPK (Jäger et al., 2007). AMPK can increase the level of NAD^+^, which results in SIRT1 phosphorylation. Activated SIRT1 deacetylates PGC-1α, thereby promoting mitochondrial biogenesis (Cantó et al., 2009). p38 MAPK cannot only regulate the expression of PGC-1α, but also phosphorylates and so activates PGC-1α (Puigserver et al., 2001). Another activator of PGC-1α is cGMP, which is upregulated due to the increase in the concentration of nitric oxide (NO) produced by NO synthases iNOS and eNOS (Nisoli et al., 2003) (Figure 1).

Phosphorylation of PGC-1α can also lead to its negative regulation. PGC-1α is phosphorylated by glycogen synthase kinase 3β (GSK3β), which inhibits PGC-1α and contributes to its intranuclear proteasomal degradation (Anderson et al., 2008). However, negative regulation of PGC-1α is carried out because of acetylation to a greater extent. GCN5 (general control of amino acid synthesis 5) is one of the most important molecules that carry out the acetylation of PGC-1α, which subsequently deacetylates by SIRT1 (Rodgers et al., 2005).

PGC-1β does not have such a wide range of action as PGC-1α, in particular it is not activated in brown fat upon cold exposure (Meirhaeghe et al., 2003). However, PGC-1β regulates the expression of NRF1 and mitochondrial biogenesis (Lin et al., 2002). The level of mitochondrial biogenesis is extremely high in transgenic mice, overexpressing PGC-1β, which even leads to the disorganization of the myofibrillar apparatus (Arany et al., 2007).

## Major Properties of the Nrf2/ARE Signaling Cascade

ARE (antioxidant response element) is a promoter element in various genes. The major activator of ARE was named Nrf2 (NF-E2-p45-related factor 2, encoded by *NFE2L2* gene) (Giudice et al., 2010). Nrf2 localizes to the cytoplasm, where it binds the specific inhibitor Keap1. In the absence of activation, Nrf2 is ubiquitinated by the E3-ubiquitin ligase-like domain of Keap1 and degraded by the 26S proteasome (Zhang and Hannink, 2003); therefore, Keap1 acts as a negative regulator of Nrf2. Nrf2 activation requited oxidation of SH-groups in Keap1 (Kansanen et al., 2013). Free Nrf2 is translocated to the nucleus, where it forms a heterodimer with the small protein Maf and binds to AREs in the target gene promoters. In most cases, these are genes coding for proteins with cytoprotective properties, e.g., antioxidant enzymes, proteins of phase II xenobiotic detoxication, and antiinflammatory enzymes. Nrf2 also regulates expression of genes involved in the regulation of redox homeostasis and a number of metabolic enzymes (Dinkova-Kostova and Abramov, 2015).

Another well-described Nrf2 repressor is GSK3β. Unlike most protein kinases, GSK3β is active under non-stress conditions and can phosphorylate Nrf2, thereby suppressing its translocation to the nucleus. However, GSK3β-induced suppression of Nrf2 can be abolished by Akt/PKB that inhibits GSK3β (Tebay et al., 2015). The E3 ubiquitin ligase Hrd1 is the third negative regulator of Nrf2, which contributes to its ubiquitination and degradation (Wu et al., 2014).

## Nrf2-Dependent Mitochondrial Biogenesis

The idea that the Nrf2/ARE signaling cascade is involved in mitochondrial biogenesis is relatively new. Only in Piantadosi et al. (2008) for the first time suggested the role of Nrf2 in the activation of mitochondrial biogenesis. The powerful incentive for the development of this field of research was the discovery of four AREs in the *NRF1* gene promoter that were capable of Nrf2 binding. The CO-stimulated production of H_2_O_2_ results in PTEN oxidation, leading to Akt/PKB activation. Akt phosphorylates and inactivates GSK3β, thereby promoting Nrf2 translocation to the nucleus. In the nucleus, Nrf2 binds to the *NRF1* promoter AREs. NRF1 activates TFAM, which is directly involved in the mtDNA replication (Figure 1).

Later, Akt phosphorylation with the following activation of the Nrf2-dependent mitochondrial biogenesis was demonstrated in heart failure treatment (Calvert et al., 2010). CO has a therapeutic effect on *Staphylococcus aureus*-caused sepsis because of the increase of expression levels of *HO-1*, both nuclear respiratory factors (*NRF1* and *NRF2*) and *TFAM*. This effect was detected neither in Nrf2-deficient nor in Akt-deficient mice (MacGarvey et al., 2012).

**Table 1:** Activation of mitochondrial biogenesis via Nrf2/ARE signal pathway.

Activation of Nrf2	Organ or cell line	Physiological condition	References
CO-stimulated H_2_O_2_ production	Heart	Non-diseased	Piantadosi et al., 2008
H_2_S	Heart	Heart failure	Calvert et al., 2010
CO	Liver	*Staphylococcus aureus-*caused sepsis	MacGarvey et al., 2012
Acetyl-L-carnitine	Brain	Hypoxia	Hota et al., 2012
Dimethyl fumarate	Brain	MPTP-induced Parkinson’s disease	Ahuja et al., 2016
Quercetin	Brain	Traumatic brain injury	Li et al., 2016
Methylene blue	Brain	Non-diseased mid-aged mice	Gureev et al., 2016
Physical exercise	Brain	6-OHDA-induced Hemiparkinsonism	Aguiar et al., 2016
Grape powder	Kidney	Non-diseased mid-aged rats	Pokkunuri et al., 2016
Physical exercise	Skeletal muscle	Non-diseased	Merry and Ristow, 2016
PNU282987	Glial cells	Non-diseased	Navarro et al., 2017
Sulforaphane	Cells LLCPK1 and PC3	Prostate cancer	Negrette-Guzmán et al., 2017
Physical exercise	Skeletal muscle	Non-diseased	Zoladz et al., 2017
*Nox4* downregulation	Lung Fibroblasts	Non-diseased	Bernard et al., 2017
Dimethyl fumarate	Peripheral blood lymphocytes	Multiple sclerosis	Hayashi et al., 2017
Acute effect of severe burn trauma	Liver	Burn trauma	Bohanon et al., 2018
β-Guanidinopropionic acid	Brain	Mid-aged mice	Gureev et al., 2018

Different Nrf2 activators were found to regulate mitochondrial biogenesis in different organs. Dimethyl fumarate disrupts the interaction between Keap1 and Nfr2 by alkylating cysteine residues in Keap1, which results in Nrf2 translocation to the nucleus and activation of mitochondrial biogenesis in multiple sclerosis (Hayashi et al., 2017) and MPTP-induced model of Parkinson’s disease (Ahuja et al., 2016). Quercetin and acetyl-L-carnitine induce Nrf2-dependent mitochondrial biogenesis in traumatic brain injury (Li et al., 2016) and hypoxia (Hota et al., 2012), respectively. Methylene blue (Gureev et al., 2016) and β-guanidinopropionic acid (Gureev et al., 2018) promote mitochondrial biogenesis in the brain of middle-aged (15-month-old) mice by inducing mild oxidative and energy stress in the mitochondria. Sulforaphane increases the number of mitochondria in cancer cells (Negrette-Guzmán et al., 2017). The α7-nicotinic acetylcholine receptor agonist PNU282987 activates mitochondrial biogenesis in cultured glial cells (Navarro et al., 2017). Moderate physical exercise induces Nrf2-dependent mitochondrial biogenesis in muscles (Merry and Ristow, 2016; Zoladz et al., 2017) and in the striatum in the 6-OHDA-induced model of parkinsonism (Aguiar et al., 2016). Nrf2-dependent mitochondrial biogenesis is activated during tissue regeneration after burn trauma (Bohanon et al., 2018). Downregulation of the *Nox4* gene expression also promotes Nrf2-dependent mitochondrial biogenesis (Bernard et al., 2017) (Table 1).

The key role of Nrf2 in mitochondrial biogenesis has also been demonstrated using Nrf2-deficient animal models. In contrast to the wild-type animals, Nrf2 activators did not increase the amounts of mitochondrial markers in the Nrf2−/− mutants (Athale et al., 2012; MacGarvey et al., 2012; Chen et al., 2014; Ahuja et al., 2016; Navarro et al., 2017). It was found that Nrf2 deficiency impairs mitochondrial biogenesis in the intestine of Nrf2−/− mice (Chen et al., 2014).

A decrease in the intensity of mitochondrial biogenesis due to impaired Nrf2 translocation to the nucleus was observed in the gentamycin-induced nephrotoxicity (Negrette-Guzmán et al., 2015), hypertonia caused by injection of low doses of lipopolysaccharide (Wu K.L.H. et al., 2016), multiple sclerosis (Hayashi et al., 2017), and in placentas of women suffering from diabetes combined with obesity (Duan et al., 2018).

## Interaction Between the Nrf2 and PGC-1α Signaling Pathways

It was found that Nrf2 translocation to the nucleus is strictly regulated by the activity of AMPK. AMPK phosphorylates Nrf2 at Ser50, which results in GSK3β inactivation. Both processes are essential for Nrf2 translocation to the nucleus (Joo et al., 2016). It was demonstrated that berberine (Mo et al., 2014), aldose reductase inhibitor fidarestat (Shukla et al., 2017), and pterostilbene (antioxidant from blueberries) (Kosuru et al., 2018) activate Nrf2, and this activation is controlled by AMPK. AMPK inhibition abolishes Nrf2 activation (Wang et al., 2016).

It is most probable that Nrf2 is indeed controlled by PGC-1α. Aquilano et al. (2013) showed that PGC-1α controls the antioxidant genes through Nrf2 activation; thus, downregulation of PGC-1α expression almost completely inhibits Nrf2 binding to the GCLC gene ARE and decreased the content of SOD2 and GCL proteins. Mice heterozygous by PGC-1α (*PGC-1*α^+/−^) exhibit downregulated expression of SOD2 because of the disrupted Nrf2 interaction with the *SOD2* gene ARE (Cherry et al., 2014). It was found that PGC-1α knockout dysregulates the Nrf2-dependent mitochondrial biogenesis (Navarro et al., 2017), although the mechanism of direct interaction between PGC-1α and Nrf2 remained unclear until recently. Choi et al. (2017) demonstrated that PGC-1α activates Nrf2 via inhibition of GSK3β. GSK3β is inactivated by p38, which is positively regulated by PGC-1α. Therefore, the PGC-1α/p38/GSK3β/Nrf2 cascade is the most probable pathway connecting these two coregulators of mtDNA transcription.

It is also possible that Nrf2 and PGC-1α form the feedback loop, i.e., Nrf2 directly influences PGC-1α expression. The PGC-1α gene promoter contains two AREs: −1723 (5′-TCTTGA**TGACATTGC**TTCTG-3′) and −226 (5′-CTGATT**TGATGGAGC**TACTT-3′) (Baldelli et al., 2013). There are data that confirm the existence of the Nrf2/PGC-1α feedback loop. Thus, the siRNA-mediated suppression of Nrf2, as well as *Nrf2* knockout, inhibit mitochondrial biogenesis and downregulate PGC-1α expression in hepatocytes (Joe et al., 2015), skeletal muscles (Whitman et al., 2013), and lungs infected with *S. aureus* (Athale et al., 2012).

There is evidence that both signaling cascades could be activated simultaneously. Metformin activates Nrf2 without involvement of the AMPK/PGC-1α axis, since inhibition of AMPK phosphorylation does not prevent metformin-induced Nrf2 activation (Prasad et al., 2017). It is, however, commonly believed that the major effect of metformin is AMPK activation due to the changes in the AMP/ATP ratio (Pawlyk et al., 2014).

Nrf2 and PGC-1α can be simultaneously activated via the Erk1/2 signaling cascade. Erk1/2 activates both Nrf2 and PGC-1α via phosphorylation of LKB1, which in turn phosphorylates AMPK (Hota et al., 2012). Interestingly, some commonly used pharmaceutical agents, e.g., erythropoietin (EPO), regulate the antioxidant defense via the Erk1/2/Nrf2/ARE axis (Genc et al., 2010; Jin et al., 2011; Wu et al., 2017). On the other hand, it was found that EPO activates mitochondria biogenesis through the Akt/eNOS/PGC-1α (Carraway et al., 2010; Qin et al., 2013) and AMPK/PGC-1α (Wang et al., 2013) pathways. Therefore, EPO can potentially activate both the Nrf2 and PGC-1α cascades. Moreover, both cascades have a common point: Akt phosphorylation can activate Nrf2 via GSK3β phosphorylation (Piantadosi et al., 2008) and directly activate eNOS (Tengan et al., 2012). Methylene blue is another compound which contributes to activation of mitochondrial biogenesis through Nrf2 by stimulation of H_2_O_2_ production (Gureev et al., 2016) and changing the relation NAD^+^/NADH, which leads to PGC-1α activation through phosphorylation by AMPK (Atamna et al., 2015).

An important study on the relation between PGC-1α and Nrf2 in mitochondrial biogenesis was published by Merry and Ristow (2016). It is known that physical exercise induces formation of reactive oxygen species (ROS) (mostly, H_2_O_2_) and nitric oxide (NO). The authors demonstrated that treatment of Nrf2^−/−^ cells with the NO and H_2_O_2_ donors failed to cause mitochondrial biogenesis activation. siRNA-mediated suppression of PGC-1α prevented NO-induced, but not H_2_O_2_-induced, activation of mitochondrial biogenesis (Merry and Ristow, 2016). Therefore, even in the case of the same event (physical exercise), mitochondrial biogenesis can be activated by different mechanisms involving different secondary messengers (Ca^2+^, NO, H_2_O_2_) (Figure 1). Both Nrf2 and PGC-1α were found to be activated simultaneously during mitochondrial biogenesis induced by exercise (Zoladz et al., 2017) and burn trauma (Bohanon et al., 2018).

Simultaneous activation of Nrf2 and PGC-1α pathways may be one of the most promising directions in gerontoprotection. In general, it relates with the fact that these two transcription factors in sum are able to regulate almost all aspects related to the functioning of mitochondria.

## Role of Nrf2 and PGC-1α in Aging

The major postulates of the aging theory were published by Harman over 50 years ago. He stated that an increase in the intensity of free-radical processes with age results in the accumulation of oxidative damage and tissue degeneration, i.e., aging (Harman, 2009). Since then, the theory expanded because of new experimental data that do not refute, but modify and refine this theory. There is widespread agreement that one of symptoms of aging is the accumulation of damaged mitochondria (López-Otín et al., 2013; Stefanatos and Sanz, 2018). For this reason, the theory of a mitochondrial “vicious cycle” became a logical continuation of Harman’s theory. Age-related increase of ROS production causes an increase of the frequency of mtDNA mutations, and they lead to ETC dysfunction, which leads to even more ROS production (Wei, 1998). Despite the logicality of this theory, it is not fully supported by experimental data (Stefanatos and Sanz, 2018). In particular, the initiation of mtDNA mutations does not cause an increase of the rate of ROS production, although it led to disruption of the functioning of the respiratory complexes, inhibition of membrane potential generation and ATP synthesis (Hiona et al., 2010). Ubiquinone depletion leads to impaired mitochondrial function, but on the contrary leads to a decrease of the rate of H_2_O_2_ production (Wang et al., 2015). These studies suggest that among all age-related mitochondrial dysfunctions, an increase of ROS production is not the most influential.

An age-related decrease of the activity of the mitochondrial respiratory chain complexes was observed almost in all studies. It is noteworthy that there is a decrease in the activity of not all components, but mainly NADH dehydrogenase (Genova et al., 1997; Navarro and Boveris, 2004; Rygiel et al., 2014) and COX (Navarro and Boveris, 2004; Ferguson et al., 2005). The decrease of the COX activity is associated with a shift of the ETC redox balance toward over-reduction. It stimulates electron leakage from ETC and subsequent ROS hyperproduction. A decrease of the complex I activity, on the contrary, may lead to a decrease of ROS production, which also has negative consequences (Stefanatos and Sanz, 2018). ROS, in particular H_2_O_2_, are important signaling molecules involved in the inactivation of negative regulators of Nrf2. An age-related decline of ROS production can lead to dysregulation of the feedback system of adaptive responses, including those by Nrf2 (Zhang H. et al., 2015).

Thus, age-related changes in ROS metabolism can cause damage to mtDNA, but mainly due to an imbalance between mitochondrial biogenesis and mitophagy. Both these processes are closely related to the Nrf2/ARE signal pathway (Piantadosi et al., 2008; Murata et al., 2015). The mtDNA mutations result from a violation of mtDNA replication and repair (Krishnan et al., 2008), as well as mitophagic dysfunction (Fivenson et al., 2017). In aging organisms, the ability of the Nrf2/ARE cascade to regulate mitochondrial biogenesis is diminished, as it has been demonstrated in rat kidneys (Pokkunuri et al., 2016) and mouse brain (Gureev et al., 2016). On the other hand, various tested chemical compounds displayed their pharmacological effects only in old rodents, which might indicate that the system of adaptive responses in young animals does not require additional external stimuli. It was found that the geroprotective effects of EPO (Wu et al., 2017), Methylene blue (Gureev et al., 2016), grape powder (Pokkunuri et al., 2016), and β-guanidinopropionic acid (Gureev et al., 2018) can be related to the Nrf2 cascade activation. Several studies demonstrated a decrease in the functional activity of Nrf2 in various organs during aging (Suh et al., 2004; Shih and Yen, 2007; Duan et al., 2009; Ungvari et al., 2011).

The SKN-1 (Skinhead-1) protein is an Nrf2 ortholog in the *Caenorhabditis elegans* nematode, the most commonly used object for studying longevity. SKN-1 is activated by various external stimuli, including oxidative stress, and induces signaling cascades regulating mitochondrial biogenesis and mitophagy resulting in the renewal of mitochondria and metabolism normalization. In combination with normalization of the mitochondria antioxidant status, coordinated regulation of mitochondrial biogenesis and mitophagy might increase the lifespan of *C. elegans* (Palikaras et al., 2015). The increase of life expectancy because of maintaining of mitochondrial homeostasis connected with the modulation of mitochondrial biogenesis and mitophagy was shown for tomatidine, activating SCN-1 (Fang et al., 2017). D-β-Hydroxybutyrate increased the activity of ETC complexes because of SCN-1 activation, which can also indirectly testify to the activation of mitochondrial biogenesis (Edwards et al., 2014). However, most of the investigations were focused on gerontoprotection via SKN-1 because of decreasing of oxidative stress and increasing of antioxidant defense. It was demonstrated for hydralazine (Dehghan et al., 2017), resveratrol and its derivatives (Fischer et al., 2017), pyrroloquinoline (Wu J.Z. et al., 2016), vitamin D3 (Mark et al., 2016), catalpol (Seo et al., 2015), curcumin (Liao et al., 2011), as well as *S*-allylcysteine, *S*-allylmercaptocysteine (Ogawa et al., 2016), and diallyl trisulfide from garlic extract (Powolny et al., 2011). SKN-1 can also be activated by mild oxidative stress induced by low arsenite doses (Schmeisser et al., 2013).

There are no studies showing the relationship between Nrf2 and the insect’s mitochondrial biogenesis at the moment. Nevertheless, there are data showing that the inhibition of GSK-3β (Castillo-Quan et al., 2016) and Keap1 loss of function (Sykiotis and Bohmann, 2008) cause lifespan extension.

The PGC-1α pathway is also involved in the process of age-related violations of mitochondrial biogenesis. Response of PGC-1α to exercise training decreases in old rats (Derbré et al., 2012). PGC-1α regulators, such as AMPK, SIRT1 and others, are also negatively regulated in the process of aging. The level of SIRT1 decreases in the microglia of old mice (Cho et al., 2015). Gong et al. (2014) showed the strongest age-dependent decrease of SIRT1 level in brain. SIRT1 levels in liver, skeletal muscle and white adipose tissue change less (Gong et al., 2014). The activation of AMPK with the help of pharmacological agents and physical exercise was blunted in the skeletal muscle of old rats (Reznick et al., 2007).

Lin et al. (2005) reported that PGC-1α structural and functional homologs were not found for several lower organisms, for example for worm, fly and yeasts. Later, dPGC-1/Spargel—a structural homolog of PGC-1α—was found for *Drosophila melanogaster* (Gershman et al., 2007). The importance of dPGC-1 in *D. melanogaster* mitochondrial biogenesis is shown in numerous studies (Tiefenböck et al., 2010; Rera et al., 2011; Mukherjee et al., 2014; Zhang F. et al., 2015; Wei et al., 2018). dPGC-1-related mitochondrial biogenesis contributes to longevity in Indy (I’m Not Dead Yet) mutant flies (Rogers and Rogina, 2014). Meldonium increased lifespan and survival rate in fly Huntington disease models via upregulation of the dPGC-1 gene (Di Cristo et al., 2018). Silencing of dPGC-1 promotes Parkinsonian phenotypes in flies (Merzetti and Staveley, 2015; Ng et al., 2017).

## Role of Nrf2 and PGC-1α in Age-Related Neurodegenerative Disease

Aging is one of the factors mediating the development of a broad range of neurodegenerative diseases. The structures of the extrapyramidal system, in particular front departments of *substantia nigra pars compacta*, are mainly affected in Parkinson’s disease (Lang and Lozano, 1998). Mitochondrial biogenesis is inhibited during Parkinson’s disease (Thomas et al., 2012). For this reason, both Nrf2 and PGC-1α are able to be targets for therapy or slowdown of Parkinson’s disease pathogenesis. It was shown that moderate physical exercises activate Nrf2-dependent mitochondrial biogenesis, which improves Parkinson’s disease symptoms in MPTP (Tsou et al., 2015) and 6-OHDA models (Aguiar et al., 2016). Dimethyl fumarate and monomethyl fumarate contributed to the treatment of MPTP-induced Parkinson’s disease by activating Nrf2-dependent biogenesis (Ahuja et al., 2016). The hereditary form of Parkinson’s disease is primarily associated with mutations in the genes encoding PARKIN and PINK1 proteins (PTEN induced kinase 1), which mediate mitophagy (Geisler et al., 2010). There is a direct connection between Nrf2 and PINK1 as Nrf2 can regulate the expression of *PINK1*, because 4 ARE regions were detected in the promoter of this gene (Murata et al., 2015). Nrf2 regulates expression of p62/SQSTM1, which acts as an adapter molecule, which then provides interaction of ubiquitinated molecules directly with the autophagosome (Jain et al., 2010). Dimethyl fumarate (Lastres-Becker et al., 2016) and the CCCP (carbonyl cyanide m-chlorophenylhydrazone) (Ivankovic et al., 2016) support p62/SQSTM1-dependent mitophagy by activating Nrf2 and contribute to Parkinson’s disease therapy. At the moment, Inosin (urate precursor—Nrf2 activator) is on the 3rd stage of clinical trials for the treatment of Parkinson’s disease (NCT02642393).

The level of PGC-1α protein decreases during Parkinson diseases in patients (Thomas et al., 2012) and in SH-SY5Y neuroblastoma cells exposed by MPP^+^ (Zeissler et al., 2016). PGC-1α null mice are much more sensitive to the neurodegenerative effects of MPTP (St-Pierre et al., 2006). The protein PARIS (ZNF746) functions as a repressor because of the KRAB domain and repress the activity of PGC-1α (Shin et al., 2011). The activity of PARIS in normal functioning neurons is repressed by Parkin (Castillo-Quan, 2011), which has ubiquitin Å3-ligase activity (Tanaka et al., 2004). Overexpression of PARIS in the absence of gene Parkin activity causes a decrease of the activity of PGC-1α-dependent mitochondrial biogenesis and a decrease of the mitochondrial number. These effects were not observed after PARIS knockout (Stevens et al., 2015). Also, compounds such as metformin and glitazone can significantly decrease the risk of Parkinson’s disease in diabetes patients via AMPK and PGC-1α (Wahlqvist et al., 2012; Brauer et al., 2015).

The main cause of Alzheimer’s disease is taupathy (hyperphosphorylation of tau protein with the formation of neurofibrillary tangles) and the accumulation of beta-amyloid plaques (Aβ). These processes are accompanied by extensive oxidative stress and neuroinflammation, so it is logical that Nrf2 is involved in these processes (Bahn and Jo, 2019). Nrf2 activators such as Puerarin (Zhou et al., 2014), Triterpenoid CDDO-methylamide (Dumont et al., 2009), β-hydroxybutyrate (Xie et al., 2015) and others contributed to lowering the level of Aβ and improving rodent cognitive parameters (Bahn and Jo, 2019). The effect of Nrf2-dependent reduction of hyperphosphorylated tau was shown for substances such as fisetin (Kim et al., 2016), benfotiamine (Tapias et al., 2018) and dimethyl fumarate (Cuadrado et al., 2018). It was shown that it is possible to reduce the level of Aβ and hyperphosphorylated Tau protein simultaneously for methylene blue (Sun et al., 2016; Zakaria et al., 2016), sulforaphane (Kim et al., 2013) and allicin (Zhu et al., 2015). Methylene blue is currently undergoing clinical trials for the treatment of Alzheimer’s disease (NCT02380573).

The role of PGC-1α during Alzheimer’s disease is very ambiguous. On the one hand, the level of *PPARGC1a* expression in postmortem tissues and therapeutic preservation of neuronal *PPARGC1a* expression is able to prevent accumulation of Aβ (Qin et al., 2009). On the other hand, *PPARGC1a* overexpression can exacerbate Aβ and hyperphosphorylated tau deposition in mice model of Alzheimer’s disease (Dumont et al., 2014).

Another neurodegenerative disease is Huntington’s disease. It is characterized by progressive choreic hyperkinesis and mental disorders caused by striatum atrophy (Joshi and Johnson, 2012). Nrf2 is a promising target for its treatment. Inhibitors of complex II (3-nitropropionic acid and malonate) can damage its structure and form the symptoms observed during Huntington’s disease. Nrf2-deficient mice are more susceptible to inhibitors of complex II, while overexpression of Nrf2, in contrast, protected neurons from the toxicity of malonate (Calkins et al., 2005). Compounds such as synthetic triterpinoids (Stack et al., 2010) and dimethyl fumarate (Ellrichmann et al., 2011) can improve the symptoms of the disease by Nrf2 activating. PGC-1α is also likely to be involved in the pathogenesis of Huntington’s disease. Mutant huntingtin represses *PPARGC1a* gene transcription (Cui et al., 2006). Huntington’s disease patients are also characterized by a decrease of PGC-1α levels (Weydt et al., 2006), but it is still not clear if this can be used as therapeutic target for the treatment of Huntington’s disease.

Thus, it can be concluded that Nrf2 activation is the most promising direction in the field of neurodegenerative disease therapy. Firstly, it provides antioxidant protection. Reducing the level of oxidative stress can significantly improve the clinical picture of diseases. Secondly, the maintenance of mitochondrial homeostasis, associated with the regulation of the number and functionality of mitochondria, can inhibit degenerative processes in the nervous tissue, significantly slowing the rate of onset of the disease. And although neurodegeneration is considered to be a severe incurable disease, it is believed that slowing down their pathogenesis is a real task, the solution of which will improve the condition of patients, prolong active longevity and delay the terminal phase of the disease as much as possible.

## Author Contributions

ES organized the article search and preliminary analysis. AG wrote the manuscript. VP revised it critically for important intellectual content. All authors contributed to a manuscript revision.

## Conflict of Interest Statement

The authors declare that the research was conducted in the absence of any commercial or financial relationships that could be construed as a potential conflict of interest.
